# 임신 여성의 적응에 관한 Giorgi의 기술적 현상학 연구

**DOI:** 10.4069/kjwhn.2020.11.26

**Published:** 2020-12-15

**Authors:** Minseon Koh, Jisoon Kim, Sukhee Ahn

**Affiliations:** 1 College of Nursing, Kyungdong University, Wonju, Korea; 1 경동대학교 간호대학; 2 Department of Nursing, Woosong University, Daejeon, Korea; 2 우송대학교 보건복지대학교 간호학과; 3 College of Nursing, Chungnam National University, Daejeon, Korea; 3 충남대학교 간호대학

**Keywords:** Adaptation, Intervention, Nursing, Phenomenology, Pregnant women, 적응, 중재, 간호, 현상학, 임신 여성

## Abstract

**Purpose:**

This descriptive phenomenological study aimed to explore the lived experience and meaning of pregnant women’s adaptation.

**Methods:**

Ten pregnant women from an ongoing Pregnant Couples' Cohort Study agreed to participate in this study. The data were collected through telephone in-depth interviews regarding what they experienced and felt about pregnancy adaptation. The qualitative data were analyzed using Giorgi’s method of descriptive phenomenology.

**Results:**

Five core situation components were extracted from the raw data, along with 12 themes and 33 focal meanings. The five core situations were 1) first recognizing the pregnancy, 2) pregnancy-related changes, 3) the upcoming birth, 4) the postpartum period, and 5) parenting. The 12 themes were as follows: *“anxiety, pressure, and embarrassment due to pregnancy,”* *“efforts to adapt to physical changes,”* *“efforts to adapt to the psychological difficulties of pregnancy,”* *“efforts to adapt to the financial burden and role changes caused by pregnancy,”* *“connecting with the fetus,”* *“adapting to a new marital relationship centering on the baby,”* *“the frustration of childbirth,”* *“fear of childbirth,”* *“postpartum care, need help with lactation planning,”* *“parenting beyond what I imagined”* *“dad’s willingness to participate in parenting,”* and *“career disconnect and consideration of workplace needs.”*

**Conclusion:**

We identified that pregnant women experience adaptation in physical, psychological, relational, and social aspects. The thematic clusters identified be used to develop nursing interventions to promote women's adaptation to pregnancy.

## Introduction

### 연구 필요성

모성 전환은 보통 임신, 출산과 산후 초기에 걸쳐서 이루어지며, 여성의 전 생애주기에서 가장 역동적인 시기이다[1]. 임신 과정 동안 여성들은 위험하고 취약한 상황에 놓여 있기도 하지만 생존하기 위한 다양한 측면의 보호적 요인을 강구하는 현상이 나타난다[2]. 임신 여성들의 경험으로부터 확인된 임신 여성 간호 실무 표준에서는 충분한 휴식과 영양 공급, 공동체 안에서의 소속감, 배우자와의 관계 북돋우기, 출산 계획에 참여하여 역량 강화하기 등 다양하고 전인적 측면의 간호를 제공하도록 제안하고 있다[3]. 또한 산후 모성 적응을 확인한 연구에서도 모성 적응은 임신에 대한 정보, 임신 계획, 사회·경제적 요인, 배우자와의 관계, 가족 및 친구의 지지 등 다양한 요인에 의해 영향을 받으며, 특히 산전 간호를 통해 자가관리를 수행하는 것이 중요함을 확인하였다[4]. 그 이유는 임신, 출산 및 육아에 대한 정보를 습득하거나 준비를 하지 않으면 예기치 않은 상황 및 이와 관련된 불안을 초래할 수 있으나, 간호사로부터 충분한 정보를 제공받고 준비를 잘하는 여성은 출산과 산후에 더욱 잘 대비하고, 안전함을 경험하며, 자신과 아기를 최상으로 돌볼 수 있기 때문이다[3].

한편 여성들은 모성 전환을 잘 인식하고 표현하지 못하는데[5], 모성 전환은 기대했던 것보다 더 스트레스가 많고, 의료인의 관심과 정보 제공, 산전 교육, 태아와의 애착 발달을 위한 기회 등 모성 전환을 위한 지지를 필요로 한다[6]. 그리고 건강한 임신 여성일지라도 의료인으로부터 세심하고 적극적이며 전문적인 정신 사회적 지지를 원하며, 더욱 세심한 관리로 임신의 변화 및 출산, 모성 역할을 준비하는 데 도움을 주기를 기대한다[7]. 그러므로 임신 여성의 적응을 촉진하기 위해 개인적 측면을 비롯한 다양한 측면에서 여성 자신의 모성 전환과 관련된 어려움을 표현하도록 돕는 전문가와의 상담이 필요하며, 이러한 간호 중재 개발이 필수적이다.

최근 국내에서 임신기 적응에 대한 연구[8,9]들은 다문화 여성을 대상으로 한 특수한 경우와 임신 여성 혹은 배우자 측면에서의 임신 및 육아 지식[10], 산전 자가 간호 행위 관련 연구[11] 등으로 개인적 측면에서 적응을 확인한 연구가 주류를 이루고 있다. 반면 개인적 측면 이외의 임신과 관련한 다른 측면이나 상황과 관련하여 임신 적응의 일반적인 구조와 의미를 확인한 연구는 찾기 어렵다. 최근 10년간 국내에서 임신 여성의 적응 양상을 탐색하고 이를 기초로 수행되는 다양한 상황과 관련된 임신 여성의 적응을 지지하기 위한 중재 연구는 찾아보기 어렵다. 그러므로 본 연구는 임부의 심층 면담을 통한 기술적 현상학 방법을 이용하여 임신 여성의 적응 현상에 대한 본질적 구조와 의미를 확인하고자 한다. 본 연구는 현시대의 임신 여성의 적응을 증진하기 위한 간호 이론 및 중재 개발을 위한 기초 자료를 제공하는 데 의의가 있다.

### 연구 목적

본 연구의 목적은 임신 여성의 적응 현상에 대한 본질적 구조와 의미를 밝히기 위함이며, 연구 질문은 *“임신 여성의 적응 양상은 어떠한가?”*이다.

## Methods

### 연구 설계

Ethics statement: This study approved by the Institutional Review Board of Chungnam National University (No. 2-1046881-A-N-01-201704-HR-008-01-04). Informed consent was obtained from the participants.

본 연구는 건강한 임신 여성의 적응 양상에 대하여 개별적이고 생생한 경험을 바탕으로 임신 여성의 적응 양상의 본질적 구조와 의미를 탐색하고 제시하기 위한 기술적 현상학(descriptive phenomenology) 연구이다.

### 연구 대상

본 연구에서는 현상학적 연구 주제인 임신 적응 현상에 맞게 임신 초기부터 말기까지의 전반적인 임신 적응과 관련된 경험을 가장 잘 드러내 줄 수 있는 임신 29주에서 임신 39주까지의 임신 여성을 연구 대상자로 선정하였다. 연구 대상자 선정기준은 제3저자가 수행하는 임산부 코호트 조사연구(임신 20주부터 산후 12주까지 총 6회 추적조사; Funding 사사 참조) 참여자 중에서, 단태아를 임신하고, 임신 합병증 및 기타 건강 문제가 없으며, 산전 우울 등 정신과 진단 경험이 없는 여성이다. 연구팀은 위 선정기준에 맞는 13명의 연구 대상자를 선별하여 연구의 목적과 취지를 설명한 후 참여 동의를 받았다. 연구 진행을 위해 대상자에게 약속을 정하고자 문자를 발송하였을 때 대상자 중 3명은 바쁜 일정으로 상담을 철회하겠다는 의사를 밝혔다. 이에 전화 상담을 약속한 시간에 진행한 연구 대상자는 10명이다.

### 자료 수집과 절차

자료 수집은 2018년 8월 21일부터 2019년 4월 26일까지 본 연구자와 연구 참여자의 전화를 통한 심층 상담으로 이루어졌다. 전화 상담을 선택한 이유는 임신 3기 참여자의 이동의 불편함 및 안전을 고려하였기 때문이다. 또한 전화 상담은 익명성이 보장되어 면대면 상담보다 상대적으로 참여자가 부담감을 덜 느끼고, 솔직하고 원활한 상담이 가능하며, 시간 및 거리의 제약을 줄일 수 있어 선호되는 상담 방식이다. 연구자는 전화 상담 약속을 하기 전에 상담의 목적은 임신 여성의 적응 양상을 확인하는 연구를 위한 것으로 예상 소요 시간은 40분에서 60분 정도이며, 상담 내용은 임신 중 전반적인 생활이나 기분에 대한 내용임을 밝혔다. 또한 상담에 참여하고 싶으면 상담을 원하는 날짜와 시간을 회신하도록 하는 문자 메세지를 보냈다. 본 상담에서 총 상담 시간은 임산부들의 상황과 피로도에 따라 최소 36분에서 57분 정도였다. 상담 시작 시에는 연구를 위한 녹음에 동의를 얻었고, *“요즘 어떻게 지내시나요?”*의 안부 질문을 시작으로 라포(rapport)를 형성하면서 긴장을 풀고 대화를 시작하였다. 참여자의 대답을 잘 경청하고 대답과 관련한 상황, 관계, 기분, 감정 등 심리, 건강 상태 등에 주목하며 임신과 관련하여 전반적인 생각, 느낌, 기분과 감정이 어떠한지, 최근 1주일간의 감정, 기분은 어떠하며 왜 그런지, 생활 사건, 일상생활, 배우자 및 태아와의 관계, 감정 및 어려움을 조절하는 방법에 대하여 추가적인 질문을 하면서 심층 상담을 진행하였다. 열 번째 참여자의 상담에서 의미 있는 새로운 자료가 나오지 않아 이를 포화 시점으로 보고 자료 수집을 마무리하였다.

### 자료 분석

임신 여성의 ‘임신 적응 양상은 어떠한가?’를 확인하기 위해 임신 여성 개개인이 경험한 현상의 의미와 본질에 대하여 해석보다는 기술에 충실한 기술적 현상학의 한 방법[12,13]으로 Giorgi [14,15]의 현상학적 방법에 따라 상담 자료를 분석하였다. Giorgi의 현상학적 연구 방법에서는 현상의 의미와 본질을 규명하고자 할 때 상황적 구조 진술과 일반적 구조 진술로 나눠 분석한다. 상황적 구조 진술에서는 연구 참여자들의 개별적인 상황에서의 개인적 특성이나 성향 등을 기술하고, 일반적 구조 진술에서는 연구 참여자들의 공통적 경험에 대해 구조와 의미들을 진술한다. 본 연구의 경우 임신 여성에서 임신 적응 양상들의 사례가 독특하여 대표성을 지님과 동시에 적응 양상에 대한 현상의 공통적이면서 본질적인 구조와 의미를 확인하고자 하는 목적을 지니고 있기에 Giorgi의 연구 접근을 사용하였다.

연구자는 심층 상담을 수행하여 구성한 원자료를 Giorgi가 제안한 분석절차에 따라 분석하였다[14,15]. 첫 번째 단계는 전체적 맥락과 구조의 이해하기 위해 연구 참여자들의 진술을 전체적으로 파악할 수 있도록 텍스트를 여러 번 읽는 단계로, 필사된 자료를 여러 번 정독하였다. 두 번째 단계는 연구자의 학문적 관점에서 참여자가 진술한 현상에 대한 의미 단위를 구분하는 것인데, 본 연구에서는 대상자의 진술문에서 총 476개의 의미 단위를 추출하였다. 세 번째 단계는 나누어진 의미 단위를 조합하여 주제화한 후, 주제 안에 담긴 의미 단위들을 연구자의 학문적 관심에 따라 ‘학문적 용어’로 변형시키는 단계로, 비슷한 개념을 묶어서 33개의 중심의미로 분류하였다. 마지막으로 네 번째 단계에서는 도출된 각 중심의미를 통합 및 분류하고 참여자의 관점에서 파악한 경험의 의미를 5개의 핵심상황으로 분류 및 체계화하여 상황적 구조를 기술하였다. 마지막으로 상황적 구조 기술문을 통합하여 전체 참여자의 관점에서 파악된 경험의 의미인 일반적 구조를 기술하였다.

### 연구자 준비

본 연구팀에서 제1저자는 모성간호학 석사 및 박사 과정 동안 질적 연구 분석 및 연구방법론 수업을 이수하였고, 지역 사회 여성들을 위한 임신 및 산욕기 부부 적응 및 부모 전환을 돕는 산전 교육 프로그램의 개발 및 중재, 산후 우울의 예방과 가족들의 돌봄, 중재를 제공한 경험이 있다. 또한 한국질적연구센터와 질적 연구학회주관으로 개최된 질적 연구 자료 수집 및 분석학술대회의 워크숍을 수료하여 연구자의 자질을 갖추기 위해 훈련하였다.

### 연구 타당성 확보

본 연구 결과의 타당성을 확보하기 위해 연구자는 Guba와 Lincoln [16] 및 Sandelowski [17]에 의해 제시된 질적 연구의 엄밀성 확보를 위한 기준을 준수하기 위하여 노력하였다. 첫째, 신빙성(credibility) 확보를 위해 간호대학 학부생을 연구 보조원으로 고용하여 녹음된 상담 내용을 자료의 누락, 왜곡이 없이 필사하였다. 자료 분석 과정에서 의미의 정확성 확인을 위해 전화 통화를 하여 참여자의 의견을 확인하고 반영하였다(3회). 둘째, 분석의 신뢰성을 위하여 공동 연구자들로부터 자료 분석 결과에 대한 검토와 피드백을 받았고, 3인의 연구 참여자로부터 도출된 주제의 보편성과 타당성을 확인하는 과정을 거쳤다. 셋째, 적용성(fittingness)을 충족하기 위해 목적적 표출법에 의하여 참여자를 선정하였으며, 더 이상 새로운 내용이 나오지 않는 포화상태에 이르기까지 계속 자료를 수집하고 분석하여 임신 여성들의 적응 양상에 대한 현상을 최대한 나타내고자 하였다. 또한 연구 참여자들의 일반적 특성을 제공하여 연구 결과의 적용성을 높이고자 했다. 넷째, 감사 가능성(auditability)을 확보하기 위해 Giorgi [14,15]에 의해 제시된 현상학적 자료 분석방법에 따라 충실하게 진행하였으며, 연구 시작에서 종료 시점까지 연구과정 전반에 대해 자세히 기록하였다. 분석 과정에 대해 공동 연구자 2인과 함께 검토하고 충실히 수행되었음을 확인받았다. 마지막으로 확인 가능성(conformability)을 충족하기 위해 참여자의 경험과 견해가 최대한 반영되도록 참여자들의 진술을 기술하였으며, 참여자들에게 면담 내용을 확인받았다. 또한 연구자의 편견과 선입견을 최소화하기 위하여 정규적인 분석회의(10회 이상)를 통해 참여자들의 진술에 대한 판단과 해석을 최대한 보류하였다. 확인 가능성은 신빙성, 적용성, 감사 가능성이 확립될 때 달성되었다고 보기 때문에 이 세 가지 기준을 모두 확립하였다고 판단됨에 따라 본 연구의 확인 가능성 또한 확보되었다고 보았다.

## Results

### 대상자의 일반적 정보

본 연구에 참여한 임신 여성의 연령은 27세에서 39세 사이였으며, 모두 초혼이고, 1명만 경임부였고 9명은 초임부였다. 직업 현황으로 3명은 직장을 갖고 있었고, 2명은 임신으로 인해 권고사직을 당했으며, 1명은 임신 준비를 위해 미리 사직한 상태였고, 나머지 4명은 주부였다. 7명은 임신을 계획하였고, 나머지 3명은 계획하지 않은 임신이었다(Table 1).

### 대상자의 상황적 구조 진술

본 연구 자료에서 도출된 5가지 핵심상황과 관련된 구조를 서술하고, 각 상황에서의 주제와 중심의미를 다음과 같이 제시하였다(Table 2).

#### 처음 임신을 인지한 상황과 관련된 구조

임신을 계획한 여성과 배우자는 기쁨과 감사의 긍정적 마음을 표현하기도 했지만, 임신을 계획했음에도 불안 및 부담스러움을 표현하였다. 반대로 비 계획 임신인 경우 임신 여성은 임신 사실에 대해 당황스러웠으나 남편이나 가족, 친구 등 주변 사람들의 지지로 인해 임신을 긍정적으로 받아들이게 되었다.

**주제모음 1**: 임신으로 인한 불안과 부담감, 당황스러움

▶중심의미 1: 계획적으로 임신하였음에도 걱정, 심란함, 무서움

*“(계획한 임신이었는데도) 두 번이 (유산)되니까 뭔가 더 심란하고 더 걱정이 컸는데…(참여자 6)”*, *“아기를 가져야지 마음을 먹었는데… 아직 덜 준비한 것 같은데… 생겨서 당황스러웠어요… 걱정되었어요(참여자 10).”*

▶중심의미 2: 임신으로 아기에게 아내를 빼앗길 것을 걱정하는 남편

*“아빠도 아기를 잘 돌보고 아기랑 친해져야 될 것 같아요. 안 그러면 또 자기가 왕따 같은 느낌이 들기도 하고… 또 애기 낳으면 저를 애기한테 뺏겼다 이런 식으로 생각할 수도 있을 것 같아요(참여자 2).”*, *“(남편이) 얘기해요. 힘들고 부담스럽다고… 우리 둘만의 시간이 중요한데 애기가 나오면 자기가 2순위로 밀릴까 봐 걱정을 많이 했는데 아기가 아들인 거를 알고 더 걱정을 했어요(참여자 9).”*

▶중심의미 3: 비계획 임신으로 부담, 놀람, 당황스러운 마음

*“계획 임신이 아니어 가지고 그냥 애기가 있구나, 라고 생각하다가… 애기보다는 내 일이 먼저 중요하니까, 관심도 사실 조금 부담이 되면서 잘 실감이 안 났던 거 같아요(참여자 3).”*, *“2년 후에 갖자고 되게 강하게 얘기를 했었던 건데… 임신한 것을 알았을 때는 너무 놀래고 당황해서… 아기에 대한 생각이 막 그렇게 있지는 않았던 것 같아요(참여자 4).”*

▶중심의미 4: 가족과 친구의 기쁨과 축하로 괜찮아짐

*“주변에서는 되게 좋아하셨죠. 다들 축하해주고… 남편이 그렇게 막 엄청 좋아할 거라는 기대는 안 했는데.. 생각보다 되게 좋아해 줘 가지고… 괜찮아졌던 것 같아요(참여자 4).”*, *“주변에 친구들이 막 이렇게 선물도 해주고… 진짜 내 일처럼 좋아해 주는 게 느껴지는 거예요. 내 일처럼 축하해줘 가지고… 그러니까는 되게 고마웠고… 좋아졌어요(참여자 5).”*

#### 임신으로 변화하는 상황과 관련된 구조

임신으로 신체적, 정신적, 사회적, 관계적 측면에서 변화가 일어나는 상황에서 신체적 건강 유지를 위한 건강 관리 및 정신적 적응을 위한 마인드 컨트롤, 재정적 부담과 사회적 역할 변화에 맞는 활동과 가치를 추구, 태아 및 배우자와의 관계적 측면의 변화에 적응하기 위한 적극적인 노력을 하였다.

**주제모음 1**: 신체적 변화에 적응하려는 노력

▶중심의미 1: 오랜 입덧의 고통을 홀로 견딤

*“(입덧은) 한 2–3개월 한 거 같아요. 조금 길었던 느낌이었어요. 과일은 거의 가리지 않고 제철과일을 거의 많이 먹었고, 달달한 거를 좀 많이 먹었던 거 같아요. 밥은 먹기는 했는데 거의 먹는 둥 마는 둥 했고요. 그리고 그 외에 다른 음식들은 거의 못 먹었던 것 같아요. 좀 남편이 거의 항상 입덧할 때는 옆에 있어 주면 좋은데, 없으니까 그거에 대한 서운함이 좀 많이 컸었죠(참여자 8).”*, *“입덧이 한 5개월까지는 갔던 거 같고… 입덧하고 6키로 빠지고는 아주 마르게 된 거… 입덧 심할 때는… 너무 힘들다 이렇게 살아가야 하나 너무 힘드니까… 마음도 축 가라앉고… 제가 저 나름의 방식으로 올라오려고 그러면 이걸 낮추는 그런 방법을 혼자 쓰면서 그냥 버텼구요(참여자 9).”*

▶중심의미 2: 임신 유지와 출산을 위한 건강 관리

*“…자연분만을 너무 하고 싶은데, 핸디캡을 줄여보자고… 체중 관리를 좀 했어요… 살을 너무 찌면 자연분만 힘들어지니까…(참여자 5)”*, *“초기 때는 아무래도 유산검사 해가지고 몸에 이상 있는 거 병원 다니면서 치료했고, 12주까지 배에다 매일 주사 맞았거든요…(참여자 6)”*, *“흡연은 하루에 한 갑 좀 넘게 피웠어요. 흡연이나 술을 임신하기 전부터 끊었어요. 아기를 생각한 부분이 있어서… 돈으로 못 해주니까 멀쩡하게라도 낳아줘야 되겠다 싶어서요(참여자 10).”*

▶중심의미 3: 임신에 적응하기 위해 산모교실 참여

*“산모교실은 한 다섯 번인가 가고… 산모교실에서 임신 전반에 대해서 설명을 해주시는 게 있었어요… 산모들이 궁금해하는 것들에 대해 질문도 많이 받아주고… 임신 중에 엄마가 하지 말아야 될 거랑… 그 기형하고 관련해가지고… 그때도 되게… 음… 좋았고(참여자 4)”*, *“네 산모교실 다니면서 좀 웃고 뭐… 저는 좀 많이 다녔어요. 산모교실을… 많을 때는… 일주일에 두 번씩 있거든요(참여자5).”*, *“…산전교육은 계속 받고 있어요(참여자 9).”*

**주제모음 2**: 임신으로 인한 심리적 어려움에 적응하기 위한 노력

▶중심의미 1: 임신으로 인한 부정적 감정을 억제하려는 마인드 컨트롤

*“애기 가지고서 검사 같은 거 하고 할 때, 걱정을 좀 많이 했던 것 같은데, …아기에게 안 좋다고 해서 안 하려고 노력을 했어요(참여자 2).”*, *“좋은 생각을 하려고 애쓰긴 했던 것 같아요… 전에는 일 관둔 것 때문에 스트레스가 많이 있어 가지고 계속 불안했었거든요. …이제 마인드 컨트롤이 필요했던… 이렇게 안 좋은 쪽으로 생각이 들을 때…(참여자 4)”*, *“너무 예민하니까 제가 책이라도 좀 보고 마인드 컨트롤을 해야겠다는…(참여자 7)”*

▶중심의미 2: 마음을 편하게 해주려는 가족의 지지

*“만약에 친정 어머님이라는 존재가 안 계셨다면… 어 그럼 굉장히 힘들겠죠(참여자 6).”*, *“남편도 그렇고, 저희 엄마도 혼자 있으면 더 우울하고 하니까 저희 집도 많이 와주고 저보고 나오라고 계속하고, 잡생각 안 들게 해준 거 같아요(참여자 7).”*

▶중심의미 3: 사회 망을 통한 심리적 지지 획득

*“집에만 있으면 너무 사람이 우울해지고, …일부러 산모교실 신청해서 주변에 다니기도 하고, 집 근처에서 친구를 만들어 보기도 하고, …산모교실이나 운동(요가)하면서 알게 된 분들 만나서 얘기도 하고 밥도 먹고 뭐 이렇게 되는 것 같아요(참여자 3).”*, *“...그냥 주변 친구들이나 지인분들이나, 동네 친구들이나…, 아파트에서 만난 언니 동생들 만나서 수다 떨고 하면 시간도 금방 가잖아요? 얘기하면 공감도 잘해주고, 따뜻하고... 같은 시기에 임신한 동생도 있어서 공감도 되고, 아닌 분들도 얘기도 잘 들어주시고 그러셔서…(참여자 7)”*

**주제모음 3**: 임신으로 인한 재정 부담과 역할 변화에 적응하려는 노력

▶중심의미 1: 임신·출산에 필요한 재정적 부담감

*“경제적인 거가 걱정되어서 고민을 하니까 그게 스트레스로 왔었던 거 같아요. 아기용품 같은 거를 구입하는 데 있어서도 금액도 많이 부담되고, 친구들 거랑 비교를 하게 되니까 그런 게 조금 힘들었어요(참여자 1).”*, *“그냥 틈틈이 사 놓아서 한 번에 너무 부담이 되지 않게 하려고 했는데, 예산은 크게 없고 꼭 필요한 것만 사려구요(참여자 9).”*

▶중심의미 2: 일과 아기 중에서 아기를 선택한 의식의 전환

*“제가 직장 생활을 하고 있었던 부분이기도 했고…(부모님이 아기를 생각하라고 해도)… 그래도 애기보다는 내 일이 먼저 중요하니까… 사실 부모님의 관심도 부담스러웠는데… 뱃속에서 애기 태동도 움직이는 거 막 느껴지고 그러면서 아… 내가 애기를 가지고 있구나, 라고 느껴진 거 같아요… 그러면서 제가 조금씩 더 이렇게 조심하는 게 있는 것 같아요(참여자 3).”*, *“일을 관두니까 자존감이 확 떨어지더라구요. 내가 없어진 거 같은 그런 느낌 있잖아요. 내 색깔이 없어진 느낌… (남편이) 일을 그렇게 많이 하지 않아도 아이를 키우고 있는 것만으로도 대단한 일을 하고 있는 거다. 뭐 그런 이야기들이 처음엔 안 들리다가 이제 점점 그런 믿음이 생겨서, …그냥 일을 놓고 아이를 키우는 게 낫겠다, 나중에는 그렇게 결정을…(참여자 4)”*

**주제모음 4**: 태아와 관계 맺기

▶중심의미 1: 태아의 존재를 인정하고 태동에 의미를 부여함

*“애기 임신했다고 한 후부터 태명을 지은 거 같아요. 태교 동화는 1, 2개 사가지고, 시간이 있을 때마다 한 번씩 읽어주기도 하고 그렇게 된 거죠(참여자 2).”*, *“너를 좋아한다. 건강하게만… (태어나라)”* *“…저희 대화할 때 아빠 목소리가 날 때 애기가 더 많이 움직이는 거 같아요… 오빠 목소리를 좋아하는 것 같다고 얘기하면… 그냥 의미를 계속 부여하는 거에요(참여자 9).”*

▶중심의미 2: 태아에게 엄마보다 더 적극적인 아빠

*“남편이 태아에게 관심이 되게 많은 것 같아요. 그냥 항상 그냥 집에 오고 가고 할 때 항상 인사하고 말 걸고 아기한테 배에다… 만지고, 만져보려고 하고, 갔다 올게, 다녀왔어, 나중에 나오면 아빠랑 축구하자, 아빠가 뭐 너 때문에 열심히 일하고 있어, 그런 이런저런 이야기를 막 해요… (참여자 4)”*, *“산전교실은 유익했던 거 같아요… 태담을 들려주는 방법이라던가 이런 거를 들었는데 아빠들은 모르니까 같이 들으니까 재미있어하더라고요…(참여자 7)”*

**주제모음 5**: 아기를 중심으로 새로운 부부관계 적응

▶중심의미 1: 부모 역할을 위한 동반자

*“…애기 아빠도 애기 크는 거 봐야 된다고 저는 일단 기본적으로 그거는 생각을 하고 있어 가지고 항상 같이 가거든요…(참여자 6)”*, *“저는 그냥 되도록 아빠랑 함께하고 싶어서, 주말에는 아빠랑 같이 함께하고 싶은 마음이 커서 그랬던 거 같아요. 또 아빠도 많이 알았으면 좋겠기도 하고...(참여자 8)”*, *“…병원은 항상 같이 갔어요. 다 그런 (산전)교육도…(참여자 9)”*

▶중심의미 2: 부부간에 이해와 배려로 맞춰감

*“…화가 나는 상황에서… 그냥 서로 맞춰가는 거 같아요(참여자 3).”*, *“…그냥 그건 저희 둘이 노력했던 것 같아요… 왜냐면 사실 일주일이면 7일을 싸웠는데… 조금 개선이 된 것 같아요(참여자 5).”*, *“훨씬 더 소중하게 느끼는 거 같아요. 말도 그렇고 행동이라던지, 배려도 더 심해졌고... 되게 성질이 있는데 그런 것도 자기가 다 참고…(참여자 6)”*

▶중심의미 3: 아내를 더욱 아끼고 소중히 여기는 남편

*“최대한 안 힘들게끔 해 줄라고 하는 거 같고, 정신적으로도 스트레스 안 받게 해 줄라 그러는 거 같고(참여자 3)”*, *“우울할 때 영향을 받는 거 같긴 한데 그렇게 크게 티 내고 그러진 않는 거 같아요. 같이 우울해지면 더 힘들어지니까 많이 다독이려고 하고, 좀 관심을 다른 데로 돌리려고 하고, 좀 그렇게 해줬던 거 같아요(참여자 4).”*

▶중심의미 4: 남편의 위로와 공감이 필요함

*“나는 이러한 점이 힘들었어, 이렇게 얘기를 하면, 나는 신랑한테 그거를 좀 위로를 받고 싶었는데… 내가 너보다 더 힘들다 니가 힘든 거는 아무것도 아니다 그런 식으로 얘기를 하는 경우에는 좀 서운했던 것 같아요(참여자 3).”*, *“아무래도 본인이 겪지 못한 일이다 보니까… 위로를 하지만 사실상 공감을 못 하니까 가끔 또 예민해지는 경우들이 한 두 번씩 생길 수 있잖아요? 그때는 이제 순간 욱하는 경우가 생기죠...(참여자 7)”*

#### 다가오는 출산과 관련된 상황적 구조

대부분의 여성들은 자연분만을 선호하지만 자연분만에 확신이 없으며, 부부가 함께 출산을 하기를 원하였다. 또한 임신 초기부터 분만 두려움이 지속되고, 매우 심하여 울기도 한 경우도 있었으며, 임신 초기부터 출산교육에 지속적으로 참여한 대상자는 분만 두려움이 완화되었다. 또한 배우자의 분만 두려움이 아내보다 더욱 심한 경우도 있고, 처음부터 제왕절개를 하라고 권유하기도 하였다.

**주제모음 1**: 출산에 대한 막막함

▶중심의미 1: 자연분만에 확신 없음

*“저는 아기도 건강하게 낳고 싶은데 제 몸이 망가지는 것도 원하지 않아서 계속 그런 거 고민이에요(참여자 1).”*, *“무통은 그냥 맞으려구요(참여자 3).”*, *“아직은 자연분만을 생각하고 있는데요. 조금 두려운 마음도 있고… 네 그래서 뭐 그냥 흘러가는 대로 하려구요(참여자 8).”*

▶중심의미 2: 부부가 함께 출산을 준비함

*“동영상 보면서 지금도 어떤 자세 하면 감통이 되는지 찾아보고 연습해보고 그러거든요. 그러다 보니까… 아 우리 둘이 같이 하면은 잘 할 수 있겠다 이런 생각이 들고, 신랑도 자신감 있게 얘기를 하더라구요, 할 수 있을 거라고…(참여자 5)”*, *“산전교육이라든지 출산 호흡법 이런 거는 수강 해놔서 오빠랑 같이 가요(참여자 9).”*

**주제모음 2**: 출산에 대한 두려움

▶ 중심의미 1: 임신 여성의 출산 두려움

*“(출산) 후기들 이런 거를 보면 막 내 배 위로 트럭이 하나 지나가는 것 같다, 막 그런 느낌들이 막 많이 든다고 하니까… 두렵고…(참여자 3)”*, *“초기에는… 분만에 대한 두려움이 엄청 크게 다가오더라구요. 너무 무서운 거예요… 두려움이 너무 컸었죠, 초반에… 갑자기 어느 날… 눈물이 막 나는 거예요. 이제는 아 될 대로 되라, 할 수 있을 거야, 이 생각이에요(참여자 5).”*

▶ 중심의미 2: 배우자의 출산 두려움

*“남편은 (제가) 겁도 많고 고통을 느끼는 거가 힘들다면 처음부터 제왕절개를 하라는 생각을 하고 있는데(참여자1)”*, *“제가 혹시 애기를 낳다가 죽을 수도 있다고 걱정하면서 지금도 되게 불안해해요. 그리고 오빠 같이 일하는 동료 분이 자연 분만하다가 죽었어요. 그래 가지고 불안감이 극도에 달했어요(참여자 9).”*, *“오빠도 무섭대요… 그냥... 일단 낳다가 저한테 무슨 일 있을까 봐 무섭대요(참여자 10).”*

▶중심의미 3: 출산 두려움 완화를 위한 산전 교육 참여

*“제가 일 다니고 있어서, 다음 주까지 일하고 이제 출산휴가 시작돼서 그때 한 번 병원에서 하는 거 가 보려고…(참여자 1)”*, *“부부 클래스를 이런 데… 같이 가면 좋겠어요. 출산에 대해서 클래스가 있다고 하면 참여하면 많이 도움이 될 것 같아요(참여자 4).”*, *“제가 산모교실 좀 열심히 다녔거든요. 어 출산 과정이라든지, 출산에 대한 내용을 많이 알려주다 보니까 출산에 대한 두려움이 많이 극복이 됐어요. 그리고 이제 하도 듣다 보니까 별일 아닌 거 같은 거? (참여자 5)”*

#### 산욕기 준비와 관련된 상황적 구조

산후에는 여성들은 산후조리원과 친정 엄마, 산후도우미 서비스를 받을 계획을 하였다. 또한 임신 여성들은 대부분 모유 수유를 우선으로 생각하고 있지만, 상황이나 젖이 잘 나오는 것에 따라서 하려고 하며, 모유 수유가 어렵다고 하면서 자신 없어 하였고, 꼭 해야겠다는 적극적인 태도를 보이지는 않았다.

**주제모음 1**: 도움이 필요한 산후 조리와 수유계획

▶중심의미 1: 산후조리 계획을 세움

*“저희 친정 부모님이… 두 분 다 맞벌이하시거든요. 산후조리를 할 수 있는 상황이 아니어 가지고… 조리원 2주 하고 도우미 원래 2주 했는데... 그렇게 혼자 조리하려면 힘드니까… 이모님 3주 쓰려구요(참여자 3).”*, *“산후조리원 2주 갔다가, 친정에서 한 달 있어 보고 뭐 괜찮으면 집으로 돌아오고, 아니면 뭐 한 달 더 있어야 되겠다 하면 친정엄마가 두 달까지… (참여자 4)”*

▶중심의미 2: 자신 없는 모유 수유

*“모유 수유는 하면 좋은데… 애기도 근데 될지 안 될지 모르니까… 안 되면… 못 하니까…(참여자 4)”*, *“수유는 모유 수유하고 싶은데 그것도 아직 잘 모르는 거라… 일단 어렵다고… 애기가 초반에는 빠는 힘이 부족하다 보니까… 이게 양이 그니까 생각보다 애기가 많이 안 빨면 그만큼 젖몸살도 오고 그럴 수 있는데…(참여자 5)”*

#### 육아 대책과 관련된 상황적 구조

육아에 대한 부모역할에 대해 막막함과 벅참을 느끼지만, 인터넷 검색과 산모교실 참여 등 육아 지식과 정보를 획득하였다. 아빠는 육아를 겁내지만 육아에 참여하기를 원하며, 심지어 육아를 전담하기로 결정한 아빠도 있었다. 또한 임신 여성들은 배우자가 육아에 함께 참여하고 아버지로서 역할을 하기 바랬다. 직장 여성의 경우 육아로 인한 경력 단절 및 육아휴직으로 인한 수입 감소로 경제적 어려움을 걱정하고, 전업 주부의 경우에는 독박 육아에 대해 두려워한다.

**주제모음 1**: 상상 이상으로 벅차게 다가오는 육아

▶중심의미 1: 육아 현실에 대비하지 않은 부모 역할의 막막함과 벅참

*“육아는 아직까지 생각하기에는 벅차서(참여자 1)”*, *“…그냥 여러 가지 면에서 아기를 잘 키워야 한다는 그런 여러 가지 면인 것 같아요(참여자 3)”*. *“신랑이랑 특별히 얘기는 해본 거는 없는 거 같아요. 단순히 잘해 보자 뭐 이런 식이고, 그렇게 하고 나서는 이제 정말 현실인데 그거에 대해서 사실 대비는 특별히 안 하고 있는 것 같아요(참여자 5).”*, *“저는 육아는… 그냥 상상 이상이라고 하더라구요(참여자 9).”*

▶중심의미 2: 독박 육아로 앞이 캄캄함

*“주변 산모들은 이미 아이를 낳아서 지금 키우고 있거든요. 근데 그런 산모들 보면 막 밤에 잠도 잘 못 자고 수유하느라고 막 바쁘고 그래도 그쪽 산모들은 신랑들이 있으니까 조금이라도 나을 텐데… 나는 처음에 나 혼자 키워야 되는데 잘 할 수 있을까… 뭐 그런 생각도 들고… 걱정이 되죠(참여자 3).”*, *“애기 낳고도 이제 애기 육아도 거의 혼자… 그래야 되지 않을까요? 그래서… 앞이 캄캄하죠. 잘할 수 있을 거 같다가도 오전부터 저녁까지니까 아무래도 저녁때 가면 체력이 안 될 거 같다는 생각이 들면서 힘들겠다는 생각이 들고…(참여자 8)”*

▶ 중심의미 3: 믿을 만한 정보의 부족과 산전교육의 실질적 도움

*“인터넷이나 맘 카페 이런 데에서 많이 얻어요(참여자 1).”*, *“뭐 친구들한테 뭐 가끔 물어보기도 하고(참여자 2)”*, *“뭐 임신 중 증상도… 인터넷에 검색해서 아는 정도인데… 인터넷에 검색을 자세히 하는 건 아니니까… 다 유튜브로만 봐서 우리가 알고 있는 게 잘 아는 건지 잘 가늠이 안 되긴 하는데…(참여자 4)”*, *“보건소에서도 강의가 있었거든요. 실질적으로 알려주니까는 좋았던 것도 있고…(참여자 5)”*

**주제모음 2**: 아빠의 육아 참여 의지

▶중심의미 1: 아빠들의 육아에 대한 의지와 걱정

*“아무래도 초보이다 보니까 제가 기대한 만큼은 못 따라와 주기는 하지만 그래도 싫다고는 안 하니까 그것만으로도 저는 고맙죠. 적극적으로 알겠다, 하겠다고 하니까…(참여자 8)”*, *“아기한테 되게 잘해주고 싶어 해요. 잘해주고 싶어서 베이비 마사지 애기들 육아 이런 거 하는데 좀 겁을 내기는 하는데… 노력하려고 많이 해요. 내가 (육아를) 잘하니까 자기한테 많이 알려주라고 얘기하고, 그냥 자기는 아기를 위해서 이런 거를 해줘야겠다고 생각을 하고 항상 고민하고 있기는 한데 자기는 잘 할 수 있는지 확신을 못 하겠대요. …시간이 없어서 혹시 애기가 내칠까 봐, 아빠 잘 가 이러면서 할까 봐. 그런 거 걱정해요(참여자 9).”*

▶중심의미 2: 아빠의 육아 담당

*“최근에 이제 애기가 곧 출산할 때가 되어 가지고, 역할 분담에 대한 그런 얘기를 하고 있어요. 제가 일을 하고 있으니까… 아직 오빠가 회사를 다니는 게 아니거든요. 그래서 육아를 오빠가 전담을 많이 하기로… 잘할 거 같아요. 아기도 좋아하고 좀 가정적이라서 그런 거는 걱정은 안 되는 거 같아요(참여자 1).”*, *“오빠 퇴근하고… 아기 낳으면 4시에 퇴근할 수 있어서… 이제 밤에 일하고… 또 주말에도 오빠가 보고…(참여자 10)”*

▶ 중심의미 3: 남편의 아기 돌봄과 애착 형성에 대한 아내의 바램

*“남편이 퇴근하고 나서 애기랑 놀 시간이 없으니까 목욕 같은 거는 남편이 좀 했으면 좋겠고... 목욕을 하면서 교감할 시간을 갖고… 마사지까지 하면은 당신이랑도 애착 관계도 잘 형성될 거 같고 좋겠다고 뭐(참여자 5)”*, *“잠잘 때 아빠랑 같이 자게 하고, 틈날 때 같이 있게 해주고, 수유 같은 거 할 때도 같이 먹이게 해주고… 더 의도적으로 이렇게… 해야 할 것 같아요(참여자 9).”*

**주제모음 3**: 직장 맘의 경력 단절과 육아에 대한 부담

▶중심의미 1: 직장 맘의 경력 단절의 숨 막힘과 우울

*“아이를 낳고 몇 년 동안 일을 못 하고, 복직이 어렵고 뭐 이제 이런 것들을 막 생각을 하니까, 어 되게 숨 막히더라구요(참여자 4).”*, *“우울해요… 그냥 뭐지… 이제 일을 그만둬야 한다는 거랑… 남편이 일을 그만두라고 하는 입장이라서(참여자 10)”*

▶중심의미 2: 직장의 업무 조정과 배려 필요

*“일을 좀 줄여주라고 얘기를 했고, 강의 말고 좀 사무직 이 쪽으로 바꿔 주셨으면 좋겠다 이런 식으로 말씀을 드리긴 했었는데…(참여자 4)”*, *“이제 애기 임신 확인하고 나서 좀 일을 많이 줄였어요. 근데 그거를 평소 하던 거에 반보다 더 많이 줄였어요(참여자 10).”*

#### 일반적 구조적 진술

**Figure. 1.**
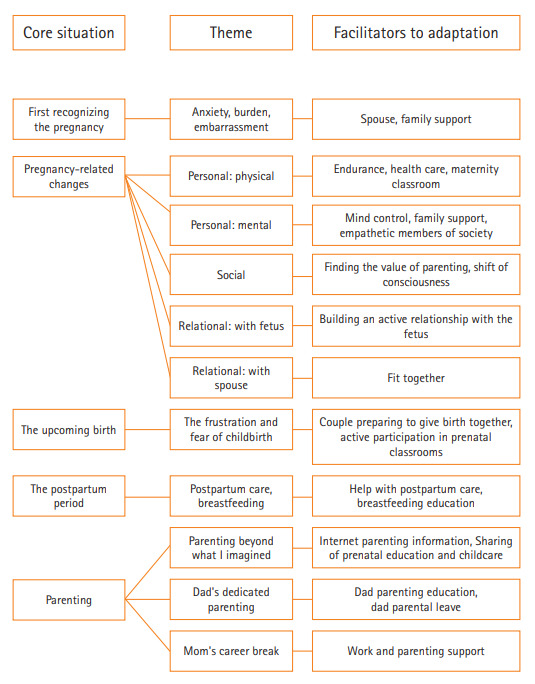
Structure of pregnant women’s adaptation.

임신 여성의 적응 양상은 임신으로 인한 시간적 흐름과 관련된 상황에 따라 나타나며, 개인적, 관계적, 사회적 측면의 노력이 필요하다. 처음 임신을 인지한 상황과 임신으로 인한 변화와 관련된 상황에서는 임부 개인 및 부부관계적 측면에서 적응을 이루려는 노력이 두드러지지만, 출산 이후의 상황에서는 임부 개인 및 부부의 노력으로도 해결할 수 없는 어려움에 대하여 사회적 지지가 필요하다. 처음 임신을 인지한 상황에서는 임신의 계획 유무에 따라 큰 차이 없이 당황스럽지만 배우자 및 가족의 지지가 도움이 된다. 임신으로 인해 신체적, 정신적, 관계적, 사회적 측면에서 변화하며, 개인 및 배우자와의 적극적인 노력으로 적응을 하고 있다. 다가오는 출산, 산후조리와 수유, 육아와 관련된 상황에서의 두려움, 불확실성, 자신감 부족에 적응하려고 노력하지만, 분만 및 모유 수유 자신감을 증가시키기 위한 산전교육, 산후조리, 육아문제 해결 등을 위한 사회적 지지가 필요하다(Figure 1).

## Discussion

본 연구는 임신 여성의 임신 적응 양상에 대한 생생한 경험을 바탕으로 본질적인 의미와 구조를 확인하였다. 참여자들의 임신 적응 양상은 개인적, 관계적, 사회적 조건 안에서 적응을 이루려는 여성 개인 및 부부의 노력이 확인되었다. 임신 적응 양상은 임신을 처음 인식하는 상황, 임신으로 인하여 변화하는 상황, 가까운 미래의 출산 및 산후, 육아 상황과 관련하여 드러났다. 임신 적응을 위해 임신 여성 개인 및 부부가 임신으로 인한 상황에 적응하기 위해 적극적으로 노력을 하고, 배우자의 부성 적응, 배우자와의 관계, 가족 지지 및 지역사회 구성원들로부터 받는 사회적 공감, 제도 등은 임신 적응에 긍정적임을 확인하였다.

임신을 계획하지 않은 경우는 물론 계획을 한 경우에도, 임신 여성뿐만 아니라 배우자도 임신을 긍정적으로 받아들이지는 않지만, 임신 과정을 겪으면서 태동을 느끼고, 부부간의, 그리고 주변 지인들의 지지 반응에 따라 점점 임신을 받아들이게 되며, 안정되고 긍정적인 태도로 변화하는 적응과정을 보여주었다. 여성뿐만 아니라 배우자도 임신기에 불안과 우울과 같은 심리적 어려움을 보이고[18], 어머니로의 적응은 시간에 걸쳐서 진행되는 과정이며 다양한 개인적, 가족적 역동성과 관련이 있다고 한 연구와 유사한 결과였다[19]. 임신을 계획하였을 때는 임신을 확인함과 동시에 임신을 긍정적으로 받아들이지만, 계획된 임신이라 할지라도 임신 여성뿐만 아니라 배우자도 자신의 건강 상태, 가족구성원 생성에 대한 준비 정도에 따라 부정적 심리상태를 나타내기도 하였다. 계획된 임신일지라도 긍정적 측면과 부정적 측면이 동시에 나타나는 것은 임신기 부부들이 기쁨과 동시에 신체적 돌봄, 자녀 양육, 경제적 필요성 등 부모 역할의 다양한 측면에서의 불안감, 부담감으로 인한 부정적 정서와 밀접한 관련성을 보인다고 한 연구 결과와 비슷하다[20]. 임신을 계획하지 않았을 경우에는 임신 초기에 부정적 심리상태를 보이며 임신을 받아들이는 데 더 오랜 시간과 배우자, 가족의 지지가 필요하였다. 이는 계획하지 않은 임신이 여성의 임신에 대한 부정적 반응으로 우울증을 유발할 수 있고, 예기치 않은 출산, 육아, 사회적 고립에 대한 두려움을 가중시킬 수 있으며, 가족 및 남편의 지지 부족으로 이어질 수 있기 때문이다[21]. 또한, 계획하지 않은 임신으로 처음에 당황하거나 양가감정을 느끼는 여성들은 산후 시기까지 심리적 고통을 호소하지만, 부부관계 적응에 따라 여성의 심리적 고통을 완화할 수 있다는 연구[22]에 따라 임신에 대한 계획뿐만 아니라 부부관계 적응을 강화함으로써 임신 초기 부정적 감정을 완화할 수 있을 것이다.

임신 기간 동안 적응을 위한 노력이 요구되는 상황은 신체적, 심리적, 사회적, 관계적인 면에서 여러 가지 양상으로 나타났다. 선행연구[21]에서도 결혼 만족도, 유산 경험, 낮은 자아존중감을 임신 중 우울의 위험요인으로 보고하였다. 임신기 부부들이 시간이 지남에 따라 심리적으로 적응하고, 남편들이 아내가 필요로 하는 부분을 함께 준비하고, 기분을 배려하고, 산전교실 같은 교육을 함께 하면서 적극적 행동 변화 및 새로운 역할을 수용하며 노력을 기울이는 것은 임신기 부부의 첫 부모 됨을 향한 적응과정이라고 하였다[20]. 본 연구 참여자들도 다양한 개인적, 관계적, 사회적 변화 상황 속에서 나름의 해결방안을 모색하고 실천하며 적응해가는 과정을 경험하는 것으로 나타났다. 즉, 참여자들은 임신으로 인한 변화 상황에 대한 해결 방법으로 개인적인 해소 방법, 태교, 남편과의 관계 개선, 산전교실 같은 전문적인 교육 참여, 지인들의 지지 등 다양한 실천적 노력을 통해 임신 기간 동안 여러 방법을 모색하고 있다. 따라서 간호사는 임신 여성의 적응과정을 촉진하기 위해 산전관리 프로그램 개발 및 적용을 통해 전문적인 지지 전략을 강화해 나아가야 할 것이다.

참여자들은 출산과 산후에 대한 나름의 계획과 해결방법을 모색하면서 임신 과정에서의 막연한 두려움을 극복하려는 모습을 나타냈다. 자연분만뿐만 아니라 제왕절개 출산에 대해서도, 임신 여성뿐만 아니라 배우자도 두려움을 호소하였다. 분만에 대한 두려움은 스트레스, 불안, 우울, 사회적 지지의 부족, 불임, 유산, 흡연, 이전 분만 경험 등 다양한 원인[23]과 관련이 있으며, 특히 임신 여성에서 불안과 배우자와의 관계, 우울이 분만 두려움의 예측요인으로 나타났다[24]. 연구 결과에서 드러난 배우자와의 지지적인 관계 유지는 분만 두려움 완화 등 임신 적응에 긍정적인 결과로 볼 수 있으며, 여자들은 부부간 격려 및 의료진의 전문적 상담, 산전교실, 부부 및 가족 출산법 등을 탐색하고 실천하는 모습을 보였다. 산후에 대해서는 산후조리원, 친정, 산후조리 서비스 이용, 모유 수유에 대한 계획을 통해 막연한 두려움을 극복하려 하였다. 이는 특히 초산모들이 어머니로의 적응기간 동안 배우자에게 가장 많이 의존하고, 배우자의 지지가 가장 중요하며, 건강 전문가의 지지[19] 및 사회적 지지는 모든 여성에게 중요하다고[25] 한 연구와 비슷한 맥락이다. 따라서 전문가가 기획하고 임신 부부가 함께 참여하는 산전교육 프로그램을 통해, 임신 과정에서 막연하게 나타나는 두려움을 해소하고 분만 두려움을 완화하며 산후의 모유 수유 자신감을 향상시키는 등의 방안을 모색하는 것도 임신 여성과 배우자에게 긍정적인 영향을 미칠 것으로 기대된다.

참여자들은 부모 역할을 부부간 과제로 여기고 임신기간 동안 지속적으로 해결하고자 고민하면서도 막막하게 생각하는 모습을 보였다. 육아, 직장 복귀로 인한 부부 역할 분배 등과 연계된 부모 역할에 대한 막막함은 부부간 당면과제로, 그 해결을 위해 구체적인 방법을 모색할 필요가 있다. 부부의 상호 지지가 임신기 부모 역할 적응에 큰 역할을 하나, 부부간 준비도의 차이가 갈등의 요인이 된다고 하였다[20]. 본 연구에서도 부모 역할에 대한 인식 차이 및 아내에 비해 직접적 신체적 경험을 하지 못하는 남편의 미흡한 준비 상태와 임신과 출산, 육아를 구체적으로 계획하지 못하는 막연함으로 인해 임신 여성이 배우자에 대한 섭섭함을 느끼는 모습이 나타났다. 또한 배우자들은 아기와의 관계에 더욱 능동적으로 참여하려고 하지만, 부모에게 요구되는 역할에 대해 전문적인 준비가 부족하다고 느꼈다[26]. 그리고 아버지로서의 시기가 다가오는 것에 대해서 계획이 모호하고[27], 아버지 역할을 위한 준비와 배우자와의 관계 변화에 대한 더 많은 지도와 지원을 원했다[28]. 이러한 연구 결과는 출산 전 부부 중심 산전 프로그램 운용 시 부부관계 증진, 효율적인 의사소통, 구체적인 육아 방법 등에 대한 전문적이고 실질적인 정보 제공 및 실천적 전략을 강화해야 함을 시사한다.

임신 여성의 적응은 임신을 계획하는 것부터 시작하며, 현재 임신의 신체적, 심리적, 사회적, 관계적 적응을 위해 개인적 노력 및 배우자와 가족, 지역사회, 의료 체계 등 사회적 지지가 도움이 되었다. 또한 미래의 출산, 산후 및 육아 계획은 현재의 임신 적응을 위해 필요한 사항으로 이를 위해 배우자의 부성 적응과 관계 적응이 필요했다. 임신 여성은 임신 기간에 신체적, 심리적, 사회적, 관계적인 면에서 적응이 요구될 때 개인적인 노력으로 해소하는 경우도 있었고, 배우자 및 가족의 지지, 지역사회 자원 및 산전교실 같은 전문가의 지지 등에 대해 적극적인 태도로 해결방법을 탐색하여 지원받고 실천함으로써 임신 적응을 위한 노력을 하였다. 그러므로 추후에는 본 연구를 통해 확인된 임신 여성의 개인적 측면 외에도 관계적 차원에서 배우자와의 관계, 가족의 지지, 배우자의 부성 적응요인을 추가하고, 사회적 차원에서는 지역사회 자조 그룹 활성화 및 산전교육 강화, 전문가 상담 등의 구성 요소를 포함할 수 있다. 따라서 임신 여성의 적응을 위해 전문인으로서 개인적, 관계적, 사회적 차원에서 적용할 수 있는 전문적 정보와 지지를 제공할 수 있는 간호 중재에 대해 전략을 수립하고 추진해야 할 것이다.

본 연구는 정상 임신 여성의 임신 과정을 통한 적응을 현상학적으로 탐색하였는데, 그 과정에서 배우자의 임신 적응에 대한 과정도 발견되었다. 그러나 본 연구는 임신 여성을 대상으로 하였기 때문에 배우자의 적응에 대한 탐색은 제한적이었다. 그러므로 추후 연구에서는 임신 여성과 배우자의 임신 적응 과정을 함께 탐색하는 현상학적 연구를 제언한다.

## Figures and Tables

**Table 1.** Characteristics of study participants (N=10)

Participant No.	Age (year)	Gestational week	Gravida	Baby’s order	Current employment	Planned pregnancy
1	27	30	1	1	Yes	Yes
2	30	38	1	1	Housewife	Yes
3	32	37	1	1	Housewife	No
4	33	36	1	1	Housewife	No
5	33	39	1	1	Housewife	Yes
6	39	29	2	2	Yes	No
7	27	31	3	1	Housewife	Yes
8	34	30	2	1	Housewife	Yes
9	36	31	1	1	Housewife	Yes
10	35	29	1	1	Yes	Yes

**Table 2.** Five core situations, themes, and focal meanings of pregnant women’s adaptation

Core situation	Theme	Focal meaning
First recognizing the pregnancy	Anxiety, pressure, and embarrassment due to pregnancy	Worried, upset, and frightened despite planning pregnancy
		Husband worried that baby will take away his wife’s love
		Burden, surprise, and embarrassment due to unplanned pregnancy
		Being okay with the joy and congratulations of family and friends
Pregnancy-related changes	Efforts to adapt to physical changes	Enduring alone the pain of long morning sickness
		Health care for pregnancy maintenance and baby delivery
		Participation in maternity classes to adjust to pregnancy
	Efforts to adapt to the psychological difficulties of pregnancy	Controlling one’s mind and active efforts to suppress negative emotions caused by pregnancy
		Family interest and support for ease of mind
		Acquiring psychological support through social network
	Efforts to adapt to the financial burden and role changes caused by pregnancy	Financial burden required for pregnancy and childbirth
		A shift in consciousness of choosing a baby between work and baby
	Connecting with the fetus	Recognizing the existence of the fetus and giving meaning to fetal movement
		Dad more active than mother towards the unborn child
	Adapting to a new marital relationship centering on the baby	Companion for parenthood
		Fit together with understanding and consideration
		A husband who cares for and values ​​his wife
		Need husband’s comfort and empathy
The upcoming birth	The frustration of childbirth	Unsure of natural childbirth
		Couple preparing for childbirth together
	Fear of childbirth	Fear of childbirth in pregnant women
		Spouse’s fear of childbirth
		Participation in prenatal education to relieve fear of childbirth
The postpartum period	Postpartum care, need help with lactation planning	Planning for postpartum care
		Breastfeeding without confidence
Parenting	Parenting beyond what I imagined	Unprepared for the reality of child-raising
		Dark thoughts about parenting alone
		Lack of reliable information and practical help for prenatal education
	Dad’s willingness to participate in parenting	Dad’s willingness and concern for parenting
		Dad taking charge of parenting
		Wife's wish for her husband to care for and become attached to the baby
	Career disconnect and consideration of workplace needs	Suffocation and depression from career breaks
		Need for coordination and consideration at work
